# How to interpret the role of SDF-1α on diabetic complications during therapy with DPP-4 inhibitors

**DOI:** 10.1186/s12933-018-0668-1

**Published:** 2018-02-02

**Authors:** Gian Paolo Fadini, Angelo Avogaro

**Affiliations:** Department of Medicine, University of Padova, Via Giustiniani 2, 35128 Padua, Italy

In a review article on this *Journal*, Milton Packer discusses the “potential confounding effect of stem-cell chemokines (SDF-1α)” on diabetic vascular complications during therapy with DPP-4 inhibitors [1]. This misconception is also part of the underlying hypothesis in another author’s recent article [2]. The purpose of this commentary is to show that such misinterpretation is not supported by data and derives from a partial and selective consideration of the literature.

The statement that SDF-1α promotes inflammation and fibrosis thereby exerting a potentially worsening effect on the course of micro- and microvascular diabetic complications derives from a flawed interpretation of available data. DPP-4 inhibition increases the concentrations of biologically active/intact SDF-1α [3], whereas in studies showing an inverse association between SDF-1α concentrations and adverse cardiovascular outcomes, total (mostly cleaved and biologically inactive) SDF-1α was measured [4, 5], the concentrations of which actually decline during therapy with a DPP-4 inhibitor [3, 6]. Several of the remaining references cited to support the author’s claim refer to genetic studies on the SDF-1 gene polymorphisms, which are not pertinent to the effect of DPP-4 inhibitors on active SDF-1α levels.

To interpret data in the right perspective, it should be noted that the best characterized physiological function of SDF-1α is the regulation of hematopoietic stem/progenitor cell kinetics [7]. As a practical example of this, plerixafor, a drug active on the SDF-1α receptor CXCR4, is clinically used to stimulate stem cell mobilization [8]. The paper by Kim et al. [9], mentioned by the author to support the claim that SDF-1α-mediated neovascularization in diabetic neuropathy in detrimental to the course of this complication, actually provides evidence that stem cell mobilization with plerixafor is a therapeutic option against diabetic neuropathy, a concept that is being tested in ongoing trials. Remarkably, DPP-4 inhibitors have been consistently shown to increase stem/progenitor cell levels in type 2 diabetes [3, 6, 10, 11]. Since reduction of stem/progenitor cells strongly predicts the development or worsening of diabetic micro- and macroangiopathy [12, 13], the effect of DPP-4 inhibitors on SDF-1α and stem cells should speculatively be protective against complications.

The author presents contrasting data on the pathophysiologic role of SDF-1α in diabetic versus non-diabetic renal disease, but fails to acknowledge studies in the literature that do not fit the proposed hypothesis, such as papers showing that SDF-1α exerts a protective action against the progression of diabetic nephropathy [14–16]. The author states that the mechanism whereby saxagliptin significantly reduced albuminuria in the SAVOR-TIMI megatrial is unknown. Quite interestingly, while definite data are certainly not available, an experimental study by Chang et al. shows that such a beneficial effect can be mediated by SDF-1α [17]. Several reviews of the literature and pooled analyses indicate a consistent favourable effects of DPP-4 inhibitors on albuminuria [18, 19]. A systematic review of preclinical and clinical studies supports that DPP-4 inhibitors can indeed improve diabetic microvascular complications [20].

Selective literature citation leads to an imbalanced perspective also in the discussion about cardiovascular outcomes trials, where the author spotlights the risk of heart failure associated with DPP-4 inhibitors, which is debated [21], but fails to mention the highly consistent risk of amputations observed with the SGLT2 inhibitor canagliflozin [22–24].

In order to avoid problems in the interpretation of the rapidly evolving literature, review articles should describe methodologies for searching databases and selecting relevant articles. Otherwise, these are personal viewpoints and not review articles based on a comprehensive consideration of all the available evidence.
